# Application of machine learning algorithms and feature selection in rapeseed (*Brassica napus* L.) breeding for seed yield

**DOI:** 10.1186/s13007-023-01035-9

**Published:** 2023-06-16

**Authors:** Masoud Shahsavari, Valiollah Mohammadi, Bahram Alizadeh, Houshang Alizadeh

**Affiliations:** 1 Department of Agronomy and Plant Breeding, College of Agriculture and Natural Resources, University of Tehran, Karaj, Iran; 2 Seed and Plant Improvement Institute, Agricultural Research, Education and Extension Organization (AREEO), Karaj, Iran

**Keywords:** Rapeseed, Machine learning, Feature selection, Selection criteria, Seed yield prediction

## Abstract

**Background:**

Studying the relationships between rapeseed seed yield (SY) and its yield-related traits can assist rapeseed breeders in the efficient indirect selection of high-yielding varieties. However, since the conventional and linear methods cannot interpret the complicated relations between SY and other traits, employing advanced machine learning algorithms is inevitable. Our main goal was to find the best combination of machine learning algorithms and feature selection methods to maximize the efficiency of indirect selection for rapeseed SY.

**Results:**

To achieve that, twenty-five regression-based machine learning algorithms and six feature selection methods were employed. SY and yield-related data from twenty rapeseed genotypes were collected from field experiments over a period of 2 years (2019–2021). Root mean square error (RMSE), mean absolute error (MAE), and determination coefficient (R^2^) were used to evaluate the performance of the algorithms. The best performance with all fifteen measured traits as inputs was achieved by the Nu-support vector regression algorithm with quadratic polynomial kernel function (R^2^ = 0.860, RMSE = 0.266, MAE = 0.210). The multilayer perceptron neural network algorithm with identity activation function (MLPNN-Identity) using three traits obtained from stepwise and backward selection methods appeared to be the most efficient combination of algorithms and feature selection methods (R^2^ = 0.843, RMSE = 0.283, MAE = 0.224). Feature selection suggested that the set of pods per plant and days to physiological maturity along with plant height or first pod height from the ground are the most influential traits in predicting rapeseed SY.

**Conclusion:**

The results of this study showed that MLPNN-Identity along with stepwise and backward selection methods can provide a robust combination to accurately predict the SY using fewer traits and therefore help optimize and accelerate SY breeding programs of rapeseed.

**Supplementary Information:**

The online version contains supplementary material available at 10.1186/s13007-023-01035-9.

## Background

Rapeseed (*Brassica napus* L.) is the second global oilseed production source after soybean, producing 13% of worldwide oil [1, 2]. The extensively cultivated double-low rapeseed, also known as canola, contains a very low amount of saturated fatty acids, palmitic C16:0 and stearic C18:0 (about 7% in total), and rich amount of unsaturated fatty acids, oleic C18:1 (about 62%), linoleic C18:2 (20%), linolenic C18:3 (10%) and eicosenoic C20:1 (1%) making it a healthy and nutritiously rich edible oil for humans [3, 4]. Owing to the energy crisis, rapeseed is also increasingly considered as a promising green energy source with minimal air pollution, and renewability [5–7]. Due to the growing demand for rapeseed oil in the food and industrial sectors, attempts to increase its yield have become inevitable [8–11].

Increasing seed yield (SY) has always been one of the major aims of breeding programs [12]. However, measuring SY in large breeding populations with thousands of genotypes is labor-intensive and time-consuming [13, 14]. Controlled by various genes and greatly affected by the environment, seed yield breeding is a highly complicated and nonlinear process [15, 16]. As a result, breeding strategies based on secondary traits (e.g., yield-related traits) that are highly linked to a primary trait enable plant breeders to efficiently identify promising lines at early stages of growth [17].

Thus far, conventional statistical methods, for instance, correlation coefficient analysis, principle component analysis (PCA), path analysis, and multiple linear regression (MLR), have been widely used in rapeseed to elucidate relationships between SY and other traits [18–21]. Nonetheless, they presume a linear relationship between the variables and are neither adequate nor comprehensive in displaying the interactions of traits and SY and would be incapable of analyzing highly nonlinear and complicated relationships between SY and other traits [22].

Machine learning algorithms have been effectively applied to optimization and prediction of many complicated biological systems [23]. The use of nonlinear machine learning algorithms in yield component analysis and indirect selection researches allows for a better understanding of nonlinear relations between yield and yield-related traits, and consequently, more precise yield prediction, which can efficiently improve breeding programs [24].

Lately, the multilayer perceptron neural networks (MLPNNs), one of the most well-known artificial neural networks (ANNs), has been widely employed for prediction and modeling complicated characteristics, such as yield, in several breeding programs and also other areas of plant sciences [17, 25]. This algorithm consists of various highly interconnected functioning neurons that can be simultaneously employed to solve a particular problem. MLPNN algorithms can also realize the intrinsic knowledge in datasets and determine the interaction between output and input variables without prior physical considerations [25, 26].

Support vector machine (SVM) is another advanced and popular machine learning algorithm with the ability to find both linear and nonlinear relationships in data [12, 27]. The benefits of employing SVMs are a large number of hidden units and better learning problem formulation, which leads to the quadratic optimization task [28]. Support Vector Regression (SVR) is the regression version of SVM and has recently been used to solve problems in agricultural and plant sciences fields [17, 25, 29–31]

Although some studies have used ANNs to predict the yield of rapeseed, they have been based on meteorological data (air temperature and precipitation) and information about mineral fertilization [4, 32, 33]. No study regarding the application of machine learning algorithms using agronomical yield-related traits has been conducted to predict the SY of rapeseed and also introducing indirect selection criteria. Furthermore, apart from MLR, ANN and SVR algorithms there are other methods such as ridge regression (RR), stochastic gradient descent (SGD) and Bayesian regression, which have not been widely used to predict SY and have remained relatively unknown to scientists in plant breeding. Therefore, in the present study, we aimed to (a) develop and optimize regression-based machine learning algorithms to predict the SY of rapeseed, (b) introduce the most important indirect selection criteria for SY of rapeseed through feature selection methods, and (c) maximize the efficiency of indirect selection for SY of rapeseed by means of finding the best combination of regression-based machine learning algorithms and feature selection methods. According to the best of our knowledge, this study is the first comprehensive report on applying a diverse range of machine learning algorithms in the field of plant breeding.

## Materials and methods

### Plant material and field experiments

Field experiments were conducted in the research farm of Seed and Plant Improvement Institute (SPII), Karaj, Iran, in the 2019–2020 and 2020–2021 growing seasons. Twenty genotypes were cultivated in the first year, and nineteen genotypes were cultivated in the second year (due to insufficient seed availability for one of the genotypes). The experiment carried out in a randomized complete block design (RCBD) with three replicates. The genotypes comprise 7 lines obtained from a pedigree experiment, a restorer line (R2000), 7 hybrids obtained from crosses between the 7 lines and R2000 and 5 cultivars (Nilufar, Neptune, Nima, Okapi and Nafis). Each plot consisted of four rows with 4 m length and with 30- and 5 cm between and within lines, respectively. Also, the distance between two plots was 60 cm. At the end of each growing season, seed yield (Kg per plot, SY) along with some important yield-related traits such as plant height (cm, PH), pods per main branch (number, PMB), pods per axillary branches (number, PAB), pods per plant (number, PP), branches per plant (number, BP), main branch length (cm, MBL), first pod height from the ground (cm, FPH), pod length (cm, PL), days to start of flowering (number, DSF), days to end of flowering (number, DEF), days to physiological maturity (number, DPM), flowering period (number, FP), thousand seed weight (g, TSW), seeds per pod (number, SP) and stem diameter (mm, SD) were recorded using 10 randomly selected plants from two intermediate rows in each plot (to prevent marginal effects) and their averages were used for training and testing datasets of algorithms.

### Data preprocessing

Data normalization is an essential preprocessing step for learning from data [34]. Moreover, when the numerical input variables have very varied scales, machine learning algorithms do not perform effectively because the algorithms could be dominated by the variables with large values [35]. To address these issues, data were normalized using Yeo-Johnson normalization method [36], and all the traits were scaled to a [0, 1] range using the Eq. (1):

1 $${X}_{scaled}=\left[\frac{\left(X-{X}_{min}\right)}{\left({X}_{max}-{X}_{min}\right)}\times \left({X}_{max}-{X}_{min}\right)\right]+{X}_{min}$$

where ${X}_{scaled}$ is the scaled value for $X$ input, ${X}_{max}$ and ${X}_{min}$ are the maximum and minimum values of$X$, respectively.

### Learning curve

**Fig. 1:**
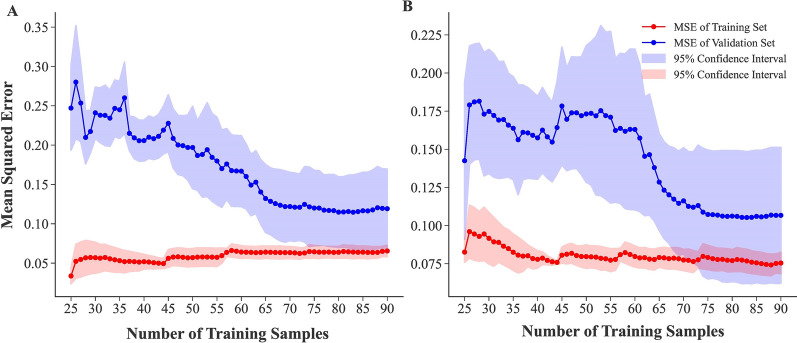
Finding the proper number of training and testing datasets using learning curve. **A**. Multiple linear regression algorithm. **B**. Ridge regression algorithm

A learning curve displays an algorithm's validation and training scores for different numbers of training samples. It is a fundamental technique to determine how much we would benefit from including extra training data, and consequently the optimal numbers of a training set [37]. To achieve this, different number of samples (from 25 to 90) were entered into MLR and ridge regression algorithms as the training set. In order to evaluate each training sample number, a 5-folds cross-validation was implemented, and then mean and 95% confidence interval of mean square errors (MSEs) were calculated in both training and validation sets. The training and the validation scores in both of the algorithms converge to a value that is quite low with increasing size of the training set (Fig. 1). MSE of validation sets approximately reached its lowest value in training size = 80 with a confidence interval overlap with the training set. Thus, training size = 80 is the proper size for the training set, and there is no benefit of more training data. The dataset was randomly divided into two subsets with 81 samples (70%) and 36 samples (30%) for training and testing data, respectively.

### Algorithm development

#### Multiple linear regression

Multiple linear regression (MLR) is a predictive technique based on linear and additive relationships of explanatory variables. MLR aims to describe the relationship between two or more explanatory variables and a dependent variable by assuming a linear relationship [38]. MLR algorithm was developed according to Eq. (2).

2 $$\widehat{y}={\theta }_{0}+ {\theta }_{1}{x}_{1}+{\theta }_{2}{x}_{2}+\dots + {\theta }_{n}{x}_{n}+\varepsilon$$

where $\widehat{y}$ is the predicted SY, ${\theta }_{0}$ is the bias term, $\theta$_1_–$\theta$_n_ are the coefficients of regression (aka feature weights), ${x}_{1}-{x}_{n}$ are the input features (traits), and *ε* is the error associated with the ${i}^{th}$ observation. Equation (2) can be concisely written in a vectorized form:

3 $$\widehat{y}={h}_{\theta }\left(x\right)=\theta .X={\theta }^{T}X$$

where ${\theta }^{T}$ is the transpose of the algorithm’s parameter vector ($\theta$), containing the bias term ${\theta }_{0}$ and the feature weights $\theta$_1_ to $\theta$_*n*_. *X* is the feature vector, containing ${x}_{0}$ to ${x}_{0}$, with $x$ always equal to 1 and ${h}_{\theta }$ is the hypothesis function, using the algorithm parameters$\theta$. The error of the algorithm is:

4 $$E\left(X, {h}_{\theta }\right)=\frac{1}{m}\sum_{i=1}^{m}{\left( {\theta }^{T}{X}^{(i)}-{y}^{(i)} \right)}^{2}$$

where $E\left(X, {h}_{\theta }\right)$ is the error, $m$ is the number of samples, and ${\theta }^{T}{X}^{(i)}$ and ${y}^{(i)}$ denote the predicted and actual amounts of SY for the ${i}^{th}$ sample, respectively.

#### Ridge regression

Ridge regression (RR) is a regularized version of MLR. Compared to MLR, RR algorithm has an additional L2 regularization term equal to $\alpha \frac{1}{2}\sum_{j=1}^{n}{\theta }_{j}^{2}$ where $\alpha$ is a non-negative hyperparameter that controls the regularization strength. The L2 regularization term is added to the error function and forces the learning algorithm to not only fit the data but also keep the algorithm weights as small as possible [35].

#### Stochastic gradient descent

Stochastic gradient descent (SGD) employs approximate gradients computed from subsets of the training dataset to update the parameters in real-time. The major advantage of utilizing this strategy is that many of the feature weights will become zero throughout training. Another benefit is that it enables us to apply the L1 regularization, bypassing the need to update the weights of features that are not used in the current sample, resulting in substantially quicker training when the feature space dimension is large [39]. Equation 5 can be used to minimize the error of the SGD algorithm:

5 $$E\left(X, {h}_{\theta }\right)=\frac{1}{m}\sum_{i=1}^{m}L\left({y}_{i} , f({x}_{i})\right)+ \alpha R\left(\theta \right)$$

where ${y}_{i}$ and $f({x}_{i})$ are the actual and predicted amounts of SY, respectively. $L$ is a loss function that measures the algorithm fitting or mis-fitting and $\mathrm{\alpha R}\left(\uptheta \right)$ is a regularization term that penalizes the algorithm complexity. Squared error (Eq. (6)), huber (Eq. (7)), epsilon insensitive (Eq. (8)), and squared form of epsilon insensitive are the loss functions that can be applied to SGD algorithm.

6 $${\mathrm{Squared \,Error}: L\left({y}_{i} , f({x}_{i})\right)}^{2}=\frac{1}{2}{\left({y}_{i}- f({x}_{i})\right)}^{2}$$

7 $${\text{Huber: is equal to MLR's cost function when }}\left| {y_{i} } \right. - \left. {f{\text{(}}x_{i} {\text{)}}} \right| \le \,\varepsilon \,{\text{and L (y}}_{i} ,f(x_{i} )) = \varepsilon \left| {y_{i} } \right. - \left. {f(x_{i} )} \right| - \frac{1}{2}\varepsilon ^{2} \,{\text{otherwise}}$$

8 $$\mathrm{Epsilon \,Insensitive}: L ({y}_{i} , f({x}_{i}))=\mathit{max}\left(0, \left|{y}_{i}- f({x}_{i})\right|-\varepsilon \right)$$

#### Generalized linear model

Generalized Linear Model (GLM) is an extended form of MLR which uses a link function, and also its loss function can be differently computed based on the given distribution [40–42]. $\widehat{y}$ is calculated through $\widehat{y}=f({\theta }^{T}X+{\theta }_{0})$, where $f$ is the link function.

#### Bayesian ridge regression

Using Bayesian theory in linear regression helps an algorithm avoid overfitting and also leads to automatic methods of determining algorithm complexity using the training dataset alone [42]. Bayesian ridge regression (BRR) is similar to the RR method, except that BRR has an additional noise precision parameter ($\lambda$) other than $\alpha$. Both $\alpha$ and $\lambda$ are estimated concurrently when the algorithm is fitting, and their priors are selected from the gamma distribution. The probabilistic model of $y$ is:

9 $$p\left({y}^{(i)}|{X}^{(i)}, \theta , \alpha \right)=N\left({y}^{\left(i\right)}|{\theta }^{T}{X}^{\left(i\right)}, \alpha \right)$$

and Gaussian prior of coefficients $\theta$ is:

10 $$p\left(\theta , \lambda \right)=N(\theta |0, {\lambda }^{-1}I)$$

A comprehensive description of Bayesian regression can be found in [42, 43].

#### Automatic relevance determination

Automatic relevance determination (ARD) (aka relevance vector machine) was first introduced by [44] and typically results in algorithms that are sparser, which allows for quicker performance on testing dataset while preserving the same generalization error. Similar to BRR, ARD is also based on Bayesian theory with the difference that each coefficient ${\theta }_{i}$ can itself be obtained from a Gaussian distribution, centered on zero and with a precision ${\lambda }_{i}$:

$$p\left(\theta , \lambda \right)=N\left(\theta |0, {A}^{-1}\right)$$

where $A$ is a positive definite diagonal matrix with a diagonal equal to: $\lambda =\left\{{\lambda }_{1}, \dots , {\lambda }_{n}\right\}$. More information on developing an ARD algorithm is available in [44, 45].

#### Support vector regression

In linear support vector regression (LSVR) we aim to minimize the Eq. (11):

11 $$\underset{\theta ,b}{\mathit{min}}\frac{1}{2}{\theta }^{T}\theta +C\sum_{i=1}max(0,\left|{y}^{(i)}-\left({\theta }^{T}\varnothing \left({x}^{(i)}\right)+b\right)\right|-\epsilon )$$

where $b$ represents bias, $C$ is regularization parameter and $\varnothing$ is the loss function (epsilon insensitive and squared epsilon insensitive can be applied).

Epsilon support vector regression (ESVR) is another form of SVR employed in this study. ESVR should be trained in such a way that the following statement would be minimized:

$$\underset{\theta , b,\zeta ,{\zeta }^{*}}{\mathrm{min}}\frac{1}{2}{\theta }^{T}\theta +C\sum_{i=1}^{m}{(\zeta }_{i}+{\zeta }_{i}^{*})$$

$${\mathrm{subject \,to }\,y}_{i}-{\theta }^{T}\varnothing \left({x}^{(i)}\right)-b\le \epsilon +{\zeta }_{i} ,$$

$$-\left({y}_{i}-{\theta }^{T}\varnothing \left({x}^{(i)}\right)-b\right)\le \epsilon +{\zeta }_{i}^{*},$$

12 $${\zeta }_{i}, {\zeta }_{i}^{*}\ge 0, i=1,\dots ,m$$

**Fig. 2:**
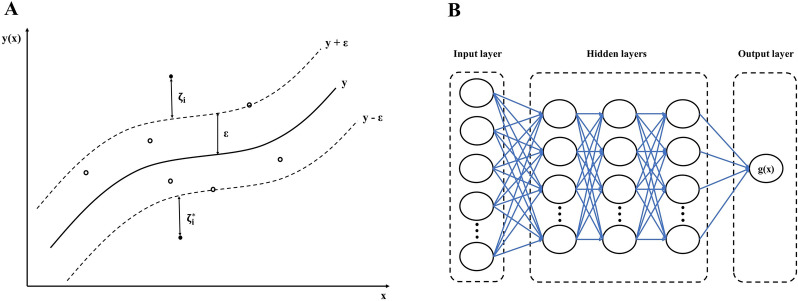
The schematic view of **A**. SVR, and **B**. MLPNN algorithms

In this case, we penalize samples whose predictions are at least $\epsilon$ off from their real target. In accordance with whether or not their predictions are placed above or below the $\epsilon$ tube, these samples penalize the objective by ${\upzeta }_{\mathrm{i}}$ or ${\upzeta }_{\mathrm{i}}^{*}$ (Fig. 2A). As having high dimensional data causes complex computational possess, it is usually more advantageous to apply the dual problem to reduce the features from *N* to *S*. The dual problem is:

$$\underset{\alpha ,{\alpha }^{*}}{\mathrm{min}}\frac{1}{2} {\left(\alpha -{\alpha }^{*}\right)}^{T}Q\left(\alpha -{\alpha }^{*}\right)+\epsilon \sum_{i=1}^{m}\left({\alpha }_{i}+{\alpha }_{i}^{*}\right)+\sum_{i=1}^{m}{y}^{(i)}\left({\alpha }_{i}-{\alpha }_{i}^{*}\right)$$

$${\mathrm{Subject to }e}^{T}\left(\alpha -{\alpha }^{*}\right)=0$$

13 $$0\le {\mathrm{\alpha }}_{\mathrm{i}},{\alpha }_{i}^{*}\le \mathrm{C},\mathrm{ i}=1,\dots ,\mathrm{m}$$

where $e$ is the vector of all ones, $Q$ is a n by n positive semidefinite matrix, and ${Q}_{is}=K\left({x}_{i},{x}_{s}\right)$ is the kernel function. Here training vectors are implicitly mapped into a higher (maybe infinite) dimensional space by the function $\varnothing$. Equation (14) shows the estimation function of ESVR algorithm.

14 $$\sum_{\mathrm{i}=1}^{\mathrm{m}}\left({\mathrm{\alpha }}_{\mathrm{i}}^{*}-{\mathrm{\alpha }}_{\mathrm{i}}\right)\mathrm{K}\left({\mathrm{x}}_{\mathrm{i}},\mathrm{x}\right)+\mathrm{b}$$

Different kernel functions Eqs. (15), (16), (17), and Eq. (18)) can be employed to ESVR algorithm.

15 $$\mathrm{Linear}: K\left({x}_{i},{x}_{s}\right)={x}_{i}^{T}{x}_{s}$$

16 $$\mathrm{Radial Basis Function }(\mathrm{RBF}): K\left({x}_{i},{x}_{s}\right)=\mathit{exp}\left(-\gamma {\left|\left|{x}_{i}-{x}_{s}\right|\right|}^{2}\right)$$

17 $$\mathrm{Sigmoid}: K\left({x}_{i},{x}_{s}\right)=\mathit{tan}h\left(\gamma {x}_{i}^{T}{x}_{s}+r\right)$$

18 $$\mathrm{Polynomial}: K\left({x}_{i},{x}_{s}\right)={(\gamma {x}_{i}^{T}{x}_{s}+r)}^{d}$$

where $\upgamma$ and $r$ are hyperparameters, and $d$ specifies the degree of the polynomial kernel function. Nu-Support Vector Regression (NuSVR) adopts a similar approach to ESVR with an additional Nu hyperparameter which controls the number of support vectors.

#### Multilayer perceptron neural network

The MLPNNs, one of the most well-known forms of ANNs, comprise an input layer, one or more hidden layers, and an output layer (Fig. 2B). A MLPNN algorithm uses Eq. (19) as loss function, which should be minimized through the training process.

19 $$\mathrm{Loss}\left(\widehat{y},y,\theta \right)=\frac{1}{2m}\sum_{i=0}^{m}{({\widehat{y}}^{(i)}-{y}^{(i)})}^{2}+\frac{\alpha }{2m}\sum_{\mathrm{j}=1}^{\mathrm{n}}{\uptheta }_{\mathrm{j}}$$

To compute the $\widehat{y}$ in the MLP with *u* neurons in the hidden layer and *z* output features, the Eq. (20) is implemented:

20 $$\widehat{y}=\sum_{j=1}^{u}{w}_{j}.g(\sum_{i=1}^{z}{w}_{ji}{x}_{i}+{w}_{j0})+{w}_{0}$$

where ${x}_{i}$ denotes the ${i}^{th}$ input feature, ${w}_{j}$ indicates the weighted input data into the ${j}_{th}$ hidden neuron, ${w}_{ij}$ shows the weight of the direct association between input neuron $i$ and the hidden neuron $j$, ${w}_{j0}$ represents the bias for node ${j}_{th}$, ${w}_{0}$ denotes the bias related to the neuron of output, and $g$ is the activation function and can be one the following items:

21 $$\mathrm{Identity}: g\left(x\right)=x$$

22 $$\mathrm{Logistic}: g\left(x\right)=\frac{1}{(1 + exp(-x))}$$

23 $$\mathrm{Tanh}: g\left(x\right)=tanh(x)$$

24 $$\mathrm{Relu}: g\left(x\right)=max(0,x)$$

### Hyperparameter optimization

In order to find the optimized values of the hyperparameters, a cross-validation method was implemented. The training dataset was first shuffled and then randomly split into train (70%), and validation (30%) sets with 150 replications, and as a result, 150 independent train-validation sets were developed. To find the optimized value of a hyperparameter in an algorithm, we first set aside the validation sets. Then we trained algorithms on train sets using a range of values for a specific hyperparameter. The trained algorithms were applied to validation sets, and the average error of each hyperparameter value was calculated. Finally, the value with the minimum amount of error was considered as the optimized value of the hyperparameter.

As hyperparameter optimization of MLPNN algorithms is computationally intensive, a five-fold cross-validation was used to optimize the hyperparameters and also the numbers of hidden layers and neurons in each hidden layer of MLPNN algorithms. We first divided the training dataset into five groups (folds). We then fitted MLPNN algorithms using four folds and then applied the algorithm to the remaining fold, and measured the error. We repeated this procedure for each of the five folds in turn. Over the 5 folds, the optimized hyperparameters were selected based on the minimum average of error.

### Algorithm performance

The algorithm performance to predict desired output was calculated using three statistical quality parameters, including root mean square error (RMSE), mean absolute error (MAE), and determination coefficient (R^2^) as follows:

25 $$RMSE=\sqrt{\frac{\sum_{i=1}^{m}{({O}_{i}-{P}_{i})}^{2}}{m}}$$

26 $$MAE=\frac{1}{m} \sum_{i=1}^{m}\left|{O}_{i}-{P}_{i}\right|$$

27 $${R}^{2}=\frac{\sum_{i=1}^{m}\left({O}_{i}-\overline{O }\right)\left({P}_{i}-\overline{P }\right)}{\sqrt{\sum_{i=1}^{m}{({O}_{i}-\overline{O })}^{2} \sum_{i=1}^{m}{({P}_{i}-\overline{P })}^{2}}}$$

where $m$ is the number of data, ${O}_{i}$ is the observed values, ${P}_{i}$ is the predicted values, and the bar denotes the mean of the feature.

### Feature selection and sensitivity analysis of input features

**Fig. 3:**
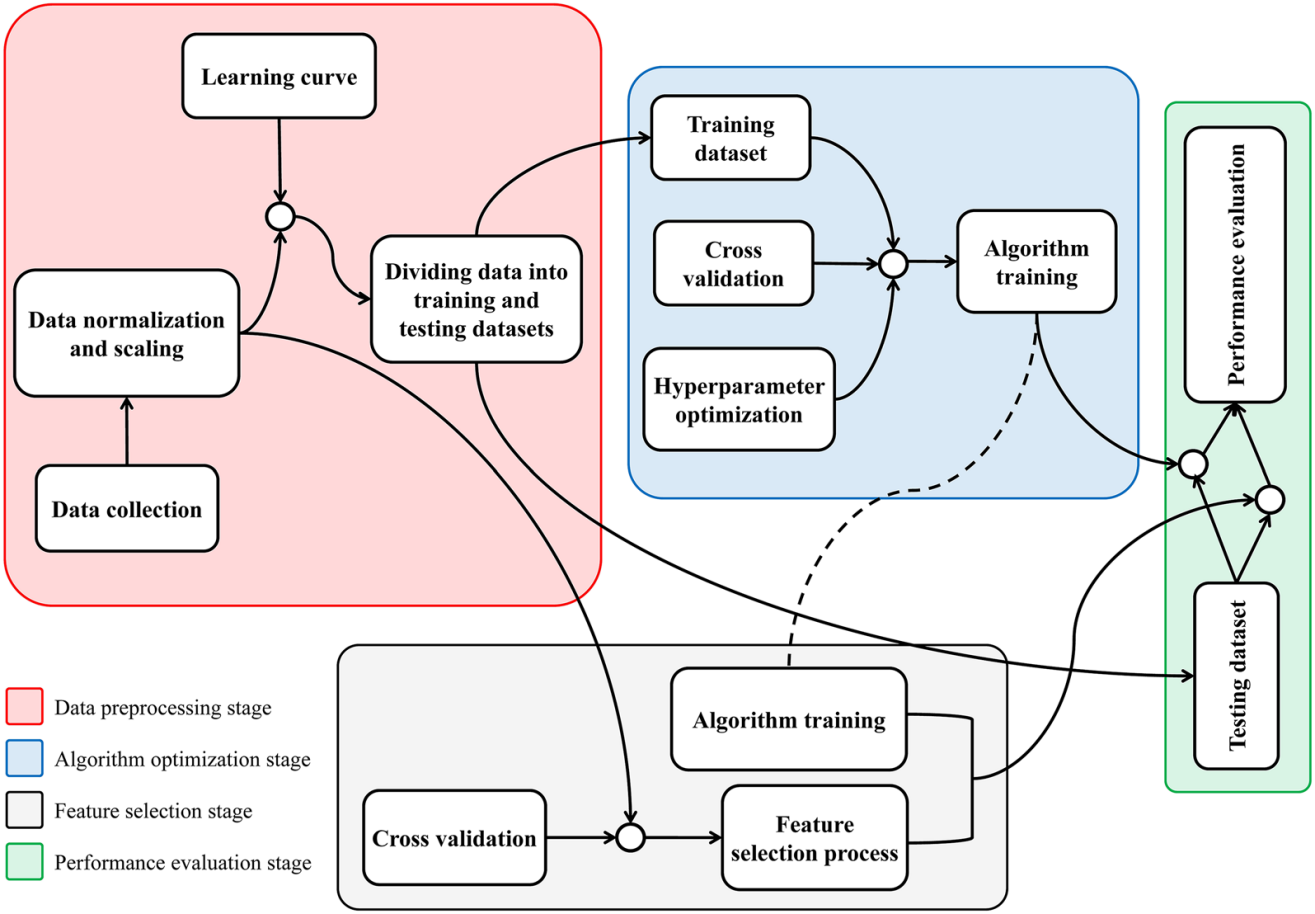
The schematic diagram of implementing and evaluating regression-based machine learning algorithms and feature selection methods

Different methods, including principle component analysis (PCA), forward selection (FS), backward selection (BS), stepwise selection (SS) [46], Pearson correlation coefficient, and lasso [47] were used to reduce the number of the yield-related traits and find the most effective traits which can justify the SY variance. Figure 3 presents a general illustration of the connection between different stages in this study. A sensitivity analysis was also performed to study the effects of various independent traits on the output and provides insight into the helpfulness of individual traits. FS, BS, and SS were conducted using caret (version 6.090) and leaps (version 3.1) packages in R (version 4.1), and other feature selection methods, algorithm development, sensitivity analysis, and visualization were conveniently implemented in Python (version 3.7.7). Trait clustering was carried out via cluster package (version 2.1.4) in R.

## Results

### Seed yield prediction using all measured traits

**Fig. 4:**
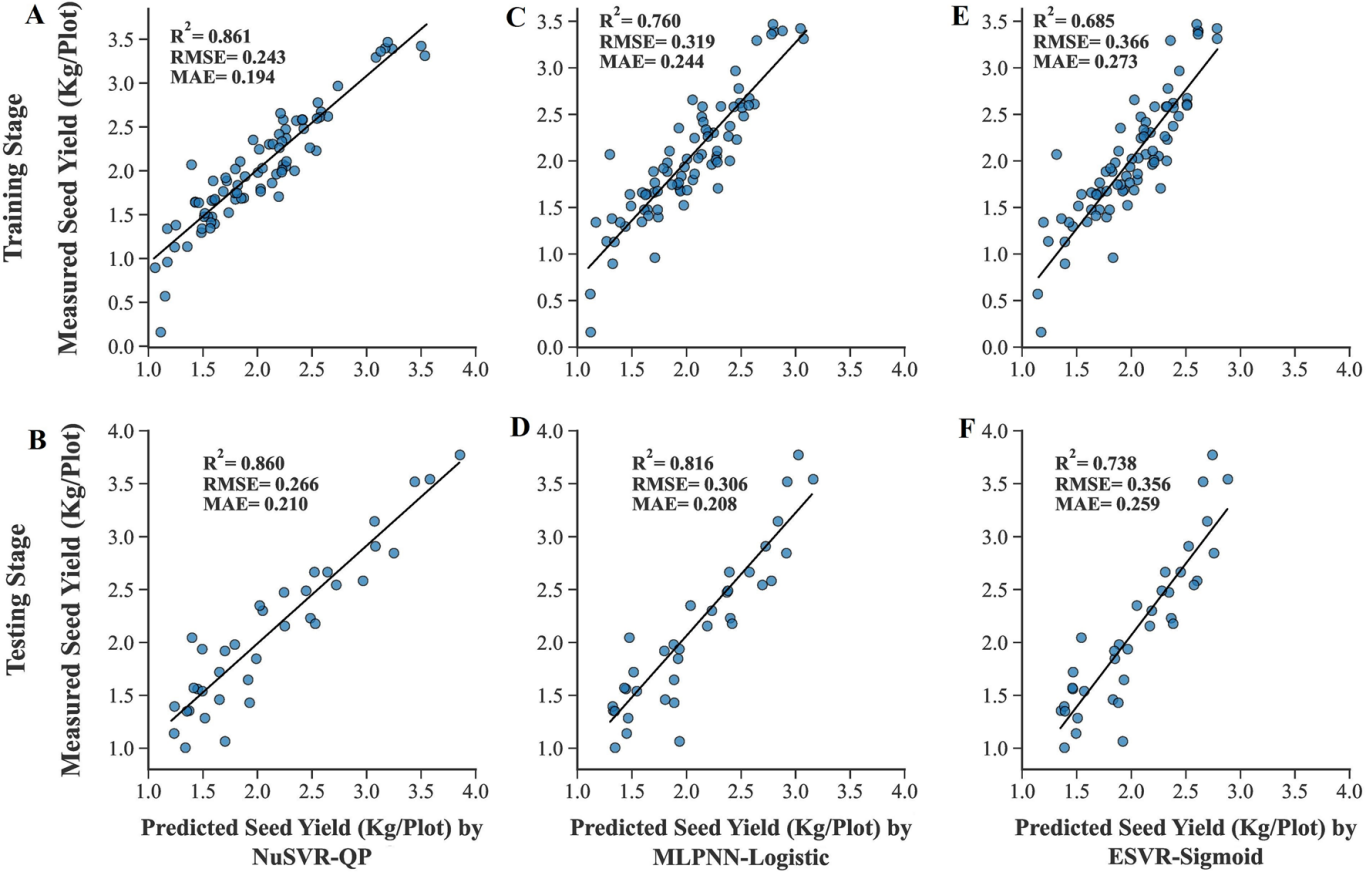
Scatter plots of measured and predicted SY of rapeseed using all measured traits as inputs. **A**, **C**, **E**. Training stage. **B**, **D**, **F**. Testing stage. *NuSVR-QP* nu-support vector regression with quadratic polynomial kernel function, *MLPNN-Logistic* multilayer perceptron neural network with logistic activation function, *ESVR-Sigmoid* epsilon support vector regression with sigmoid kernel function

**Fig. 5:**
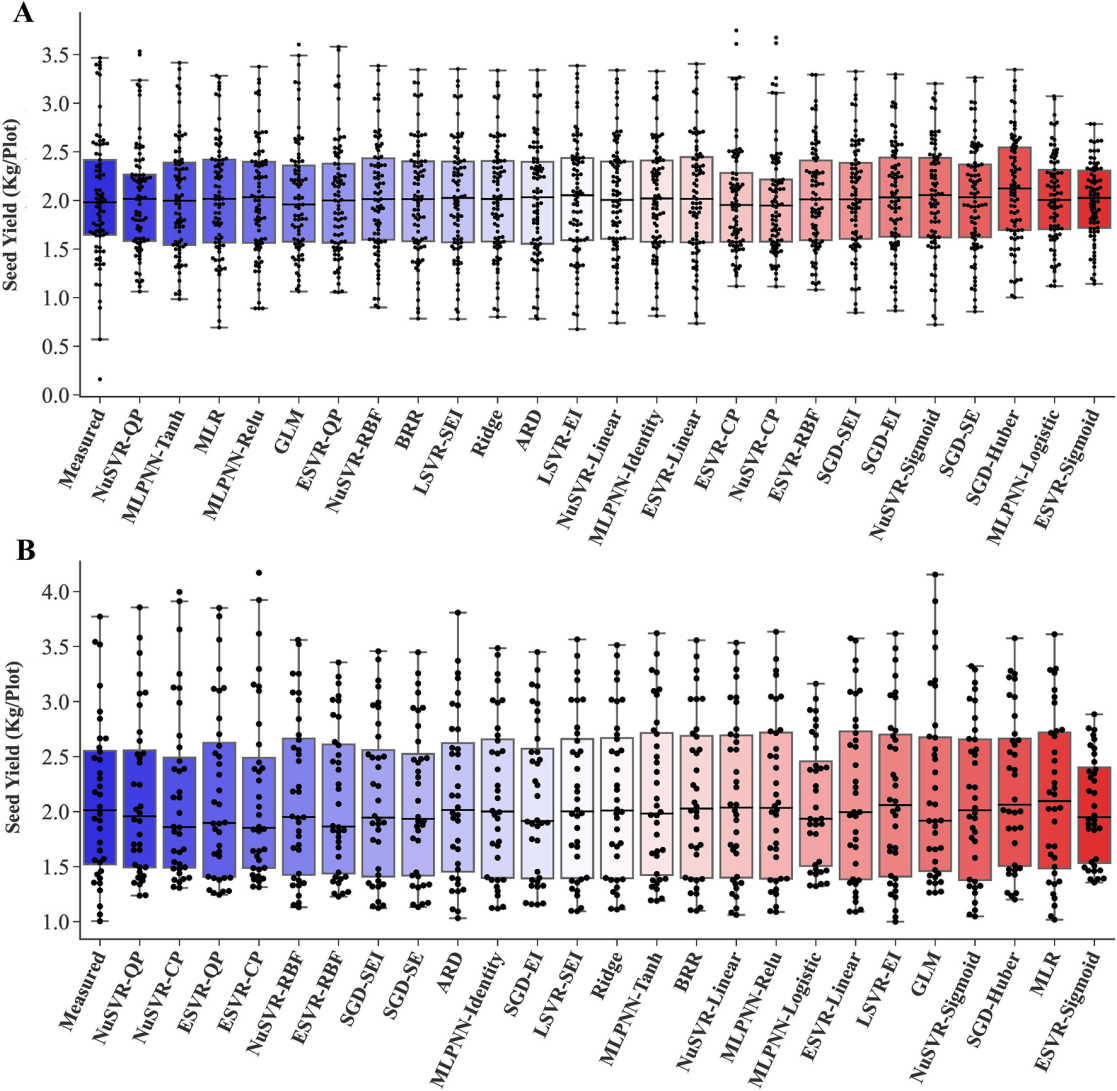
Box plots of measured and predicted SY of rapeseed using all measured traits as inputs. Algorithms are sorted based on the highest to lowest R^2^ value from left to right. **A**. Training stage. **B**. Testing stage. *MLR* multiple linear regression, *BRR* Bayesian ridge regression, *ARD* automatic relevance determination, *GLM* generalized linear model, *SGD* stochastic gradient descent, *NuSVR* nu-support vector regression, *ESVR* epsilon support vector regression, *LSVR* linear support vector regression, *MLPNN* multilayer perceptron neural network, *RBF* radial basis function, *QP* quadratic polynomial, *CP* cubic polynomial, *EI *epsilon insensitive, *SEI* squared epsilon insensitive

A total of 25 algorithms were developed and optimized to predict the SY of rapeseed. All measured yield-related traits were entered into the algorithms as inputs and their performances were evaluated using R^2^, RMSE, and MAE values (Tables 1, 2). According to the results, the least amounts of RMSE and the highest R^2^ values were achieved using the NuSVR algorithm with quadratic polynomial kernel function (NuSVR-QP) in both training and testing stages (Fig. 4A, B), followed by the MLPNN algorithm with tanh activation function (MLPNN-Tanh) and the NuSVR algorithm with Cubic polynomial kernel function (NuSVR-CP) in the training and testing datasets, respectively. The least amounts of training MAE were seen in the MLPNN algorithm with tanh and relu activation functions, respectively. MLPNN algorithm with logistic activation function (MLPNN-Logistic) had the least testing MAE value (Fig. 4D) prior to NuSVR-QP. The least a*c*curacy of the algorithms was achieved by ESVR algorithm with sigmoid kernel function (ESVR-Sigmoid) in all statistical criteria and both training and testing datasets (Fig. 4E, F), followed by MLPNN-Logistic in the training stage and MLR in the testing stage. The predicted and measured values of SY in both training and testing datasets were presented and contrasted as box plots to provide a better understanding of the data distribution and the effectiveness of algorithms to predict SY (Fig. 5).

**Table 1:** The performance of the algorithms to predict the SY of rapeseed using all measured traits

Algorithm	Kernel function /Loss function	Training			Testing		
		R^2^	RMSE	MAE	R^2^	RMSE	MAE
Multiple Linear Regression (MLR)	–	0.856	0.247	0.191	0.786	0.329	0.254
Ridge Regression (RR)	–	0.843	0.258	0.198	0.830	0.294	0.234
Bayesian Ridge Regression (BRR)	–	0.846	0.255	0.196	0.825	0.298	0.236
Automatic Relevance Determination (ARD)	–	0.842	0.259	0.205	0.834	0.290	0.227
Generalized Linear Model (GLM)	–	0.849	0.253	0.194	0.809	0.311	0.243
Stochastic Gradient Descent (SGD)	Squared Error (SE)	0.809	0.285	0.222	0.839	0.286	0.224
	Huber	0.788	0.299	0.232	0.791	0.325	0.251
	Epsilon Insensitive (EI)	0.814	0.281	0.218	0.832	0.292	0.227
	Squared Epsilon Insensitive (SEI)	0.818	0.277	0.216	0.841	0.284	0.223
Nu-Support Vector Regression (NuSVR)	Linear	0.841	0.259	0.195	0.823	0.300	0.237
	Radial Basis Function (RBF)	0.847	0.255	0.194	0.841	0.284	0.219
	Sigmoid	0.813	0.282	0.213	0.809	0.312	0.246
	Quadratic Polynomial (QP)	0.861	0.243	0.194	0.860	0.266	0.210
	Cubic Polynomial (CP)	0.826	0.271	0.210	0.851	0.275	0.227
Epsilon Support Vector Regression (ESVR)	Linear	0.836	0.263	0.204	0.815	0.307	0.242
	Radial Basis Function (RBF)	0.819	0.277	0.211	0.841	0.284	0.223
	Sigmoid	0.685	0.366	0.273	0.738	0.356	0.259
	Quadratic Polynomial (QP)	0.848	0.253	0.193	0.846	0.279	0.220
	Cubic Polynomial (CP)	0.834	0.265	0.198	0.843	0.282	0.232
Linear Support Vector Regression (LSVR)	Epsilon insensitive (EI)	0.842	0.258	0.191	0.813	0.308	0.238
	Squared Epsilon Insensitive (SEI)	0.843	0.258	0.197	0.830	0.294	0.232

*R*^2^ determination coefficient, *RMSE* root mean square error, *MAE* Mean absolute error

**Table 2:** The performance of the MLPNNs to predict the SY of rapeseed using all measured traits

Algorithm	Activation function	Best hidden layers topology	Training			Testing		
			R^2^	RMSE	MAE	R^2^	RMSE	MAE
Multilayer Perceptron Neural Network (MLPNN)	Identity	5	0.840	0.260	0.200	0.832	0.292	0.233
	Logistic	5	0.760	0.319	0.244	0.816	0.306	0.208
	Tanh	4–5–5	0.857	0.246	0.188	0.827	0.295	0.234
	Relu	2-5-4-2	0.855	0.247	0.190	0.820	0.302	0.237

*R*^2^ determination coefficient, *RMSE* root mean square error, *MAE* mean absolute error

**Fig. 6:**
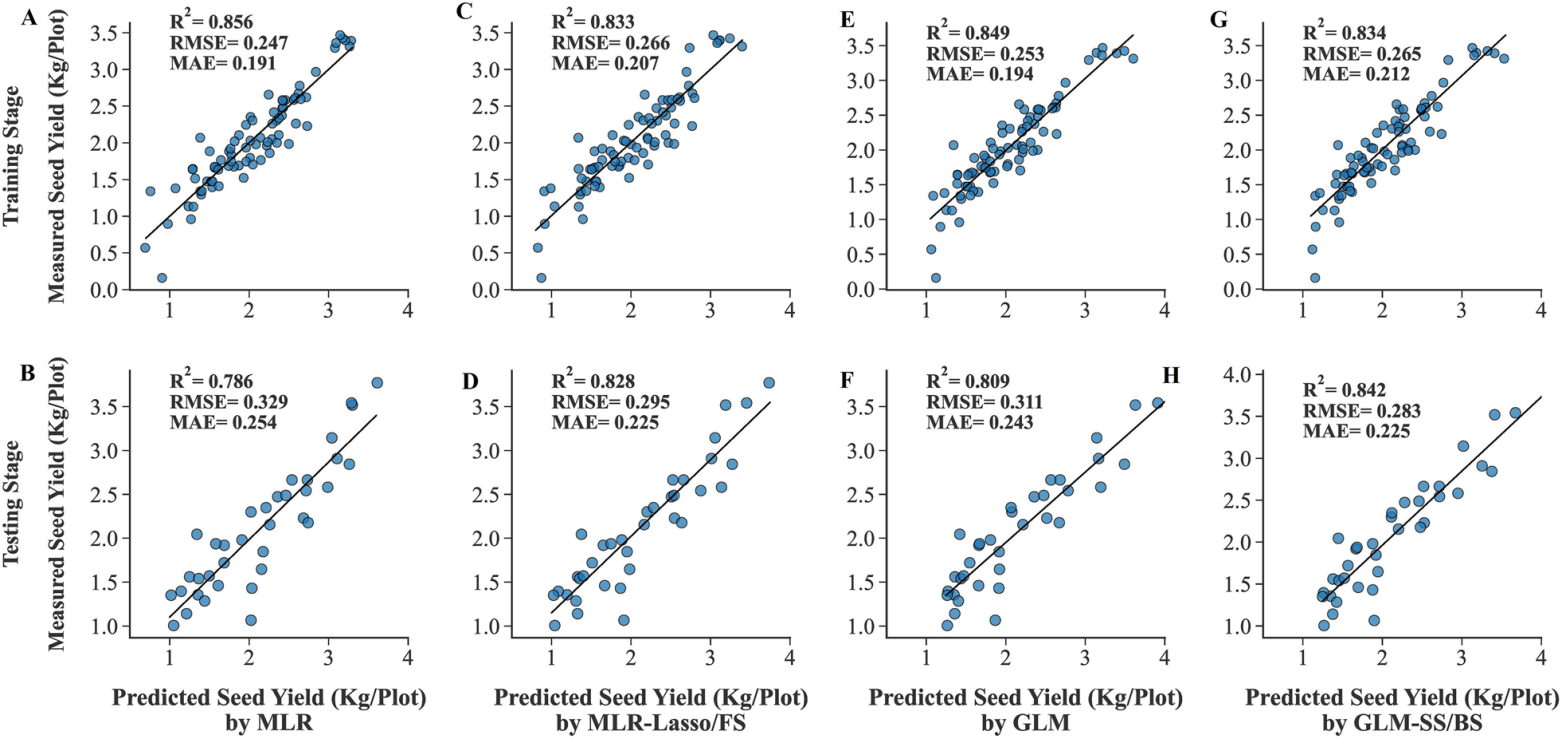
Scatter plots of measured and predicted SY of rapeseed using MLR and GLM algorithms. **A**, **C**, **E**, **G**. Training stage. **B**, **D**, **F**, **H**. Testing stage. *FS* forward selection, *SS* stepwise selection, *BS* backward selection

In the present study, the reduction of R^2^ value and the increase of RMSE and MAE amount between testing and training datasets of MLR (with R^2^_Test_–R^2^_Train_ = − 0.07, RMES_Test_–RMSE_Train_ = 0.082, MAE_Test_–MAE_Train_ = 0.063) demonstrated that MLR is the most overfitted algorithm followed by GLM algorithm (with R^2^_Test_–R^2^_Train_ = − 0.04, RMES_Test_–RMSE_Train_ = 0.058, MAE_Test_–MAE_Train_ = 0.049). It has also been shown in the scatter plot of the MLR and GLM algorithms (Fig. 6A, B, E, F) that they fit very well in the training stage; however, they have not been capable of repeating the same performance in the testing stage.

### Feature selection and SY prediction using selected traits

In order to reduce the dimensions of the data and find the most important variables in predicting SY in rapeseed genotypes, 6 different feature selection methods including Pearson correlation coefficient, principal component analysis (PCA), stepwise selection (SS), forward selection (FS), backward selection (BS), and lasso were used in this study. To avoid overfitting in the SS, FS, and BS methods, leaps and caret packages in R with a five-fold cross-validation were employed to create 10 trait subsets. The first subset included the first trait selected by each method, and in the following subsets, one trait was added to the previous trait(s). Based on the R^2^, RMSE and MAE values of the cross-validation stage, the best subsets were achieved using PP, FPH, and DPM in the SS and BS methods and PP, PH, and DPM in the FS method (Table 3).

**Table 3:** The output of stepwise selection, forward selection, and backward selection methods

Method	Most efficient subset	R^2^	RMSE	MAE
Stepwise Selection	PP, FPH, DPM	0.810	0.288	0.224
Forward Selection	PP, PH, DPM	0.816	0.281	0.219
Backward Selection	PP, FPH, DPM	0.808	0.291	0.227

*PP*: pods per plant, *FPH* first pod height from the ground, *DPM* days to physiological maturity, *PH* plant height, *R*^2^ determination coefficient, *RMSE* root mean square error, *MAE* mean absolute error

Using the ability of the lasso method to effectively reduce the number of features by giving zero coefficients to less important variables led to the Eq. (28)

28 $$SY = 0.736 + 0.608\,PH + 2.055\,PP + 0.409\,DPM$$

where the SY is seed yield, the PH is plant height, the PP is pods per plant, and the DPM is days to physiological maturity. As can be seen from the results of FS and lasso methods, both had the same traits as output.

**Fig. 7:**
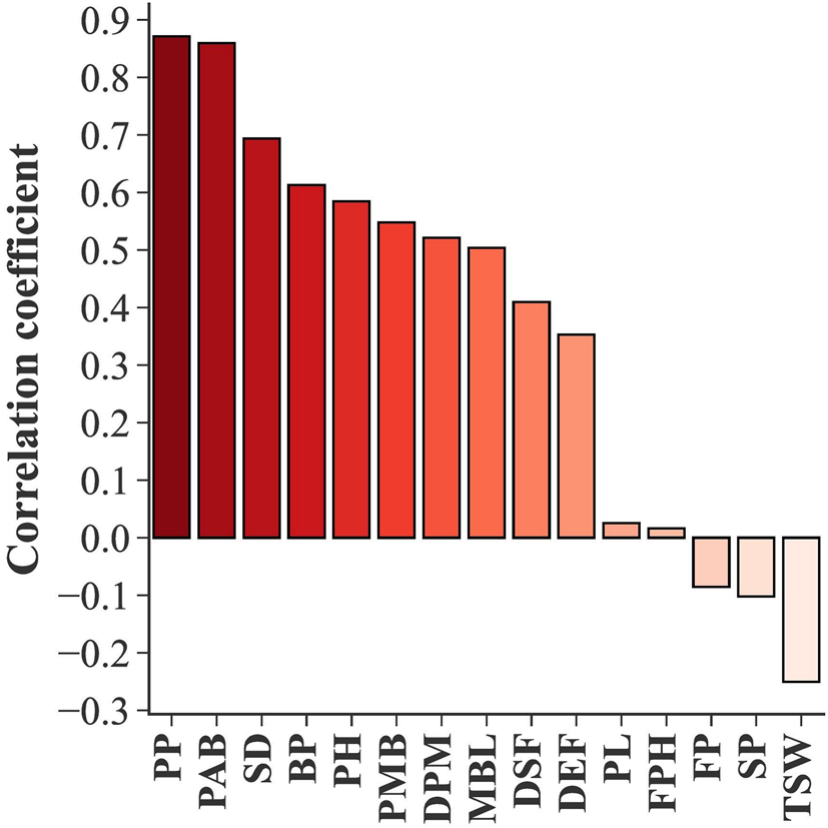
Pearson correlation coefficients of yield-related traits and seed yield in rapeseed genotypes. *PP* pods per plant, *PAB* pods per axillary branches, *SD* stem diameter, *BP* branches per plant, *PH* plant height, *PMB* pods per main branch, *DPM* days to physiological maturity, *MBL* main branch length, *DSF* days to start of flowering, *DEF* days to end of flowering, *PL* pod length, *FPH* first pod height from the ground, *FP* flowering period, *SP* seeds per pod, *TSW* thousand seed weight

Since having 3 traits in all variable selection methods could enable us to compare the methods with the same number of variable subsets, three traits were also selected in Pearson correlation coefficient and PCA methods. The results of the Pearson correlation coefficient showed that PP, PAB, and SD had the highest positive correlations with SY of rapeseed genotypes (Fig. 7). PP, PAB, and BP were the selected traits based on PCA results (Table 4).

**Table 4:** Principal component analysis of yield-related traits in rapeseed genotypes

Trait	PH	PMB	PAB	PP	BP	MBL	FPH	PL	DSF	DEF	DPM	FP	TSW	SP	SD	EVR%
PC1	0.27	0.19	0.44	0.47	0.37	0.21	0.01	0.02	0.25	0.31	0.11	0.05	− 0.16	− 0.02	0.30	35.64
PC2	0.23	− 0.02	− 0.23	− 0.24	-0.44	0.12	0.12	− 0.07	0.36	0.51	0.16	0.14	− 0.39	0.07	− 0.12	16.50

*PC* principal component, *PH* plant height, *PMB* pods per main branch, *PAB* pods per axillary branches, *PP* pods per plant, *BP* branches per plant, *MBL* main branch length, *FPH* first pod height from the ground, *PL* pod length, *DSF* days to start of flowering, *DEF* days to end of flowering, *DPM* days to physiological maturity, *FP* flowering period, *TSW* thousand seed weight, *SP* seeds per pod, *SD* stem diameter, *EVR* explained variance ratio

**Fig. 8:**
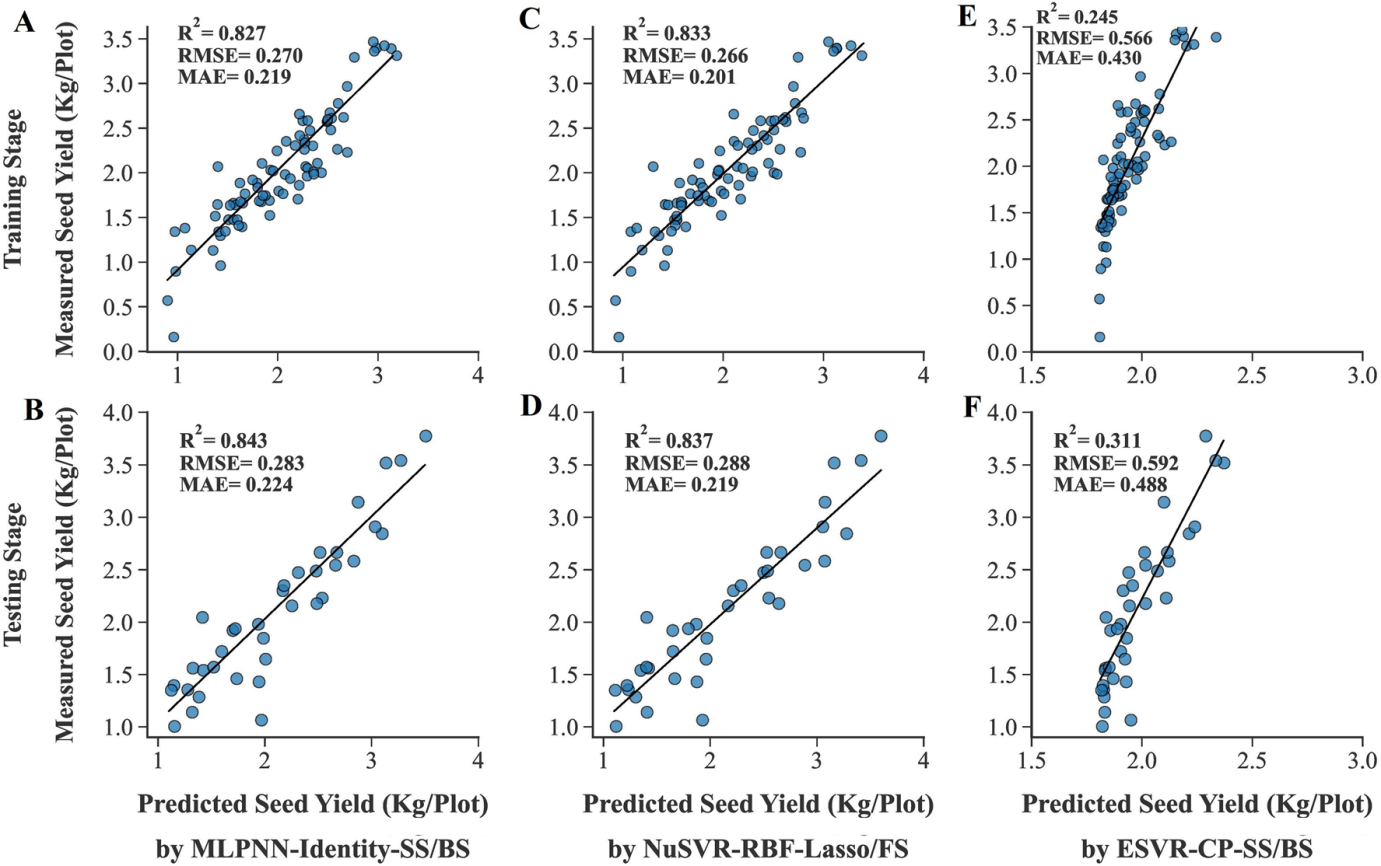
Scatter plots of measured and predicted SY of rapeseed using selected traits as inputs. **A**, **C**, **E**. Training stage. **B**, **D**, **F**. Testing stage. *MLPNN-Identity* multilayer perceptron neural network with identity activation function, *NuSVR-RBF* nu-support vector regression with radial basis function kernel function, *ESVR-CP* epsilon support vector regression with cubic polynomial kernel function, *SS* stepwise selection, *BS* backward selection, *FS* forward selection

**Fig. 9:**
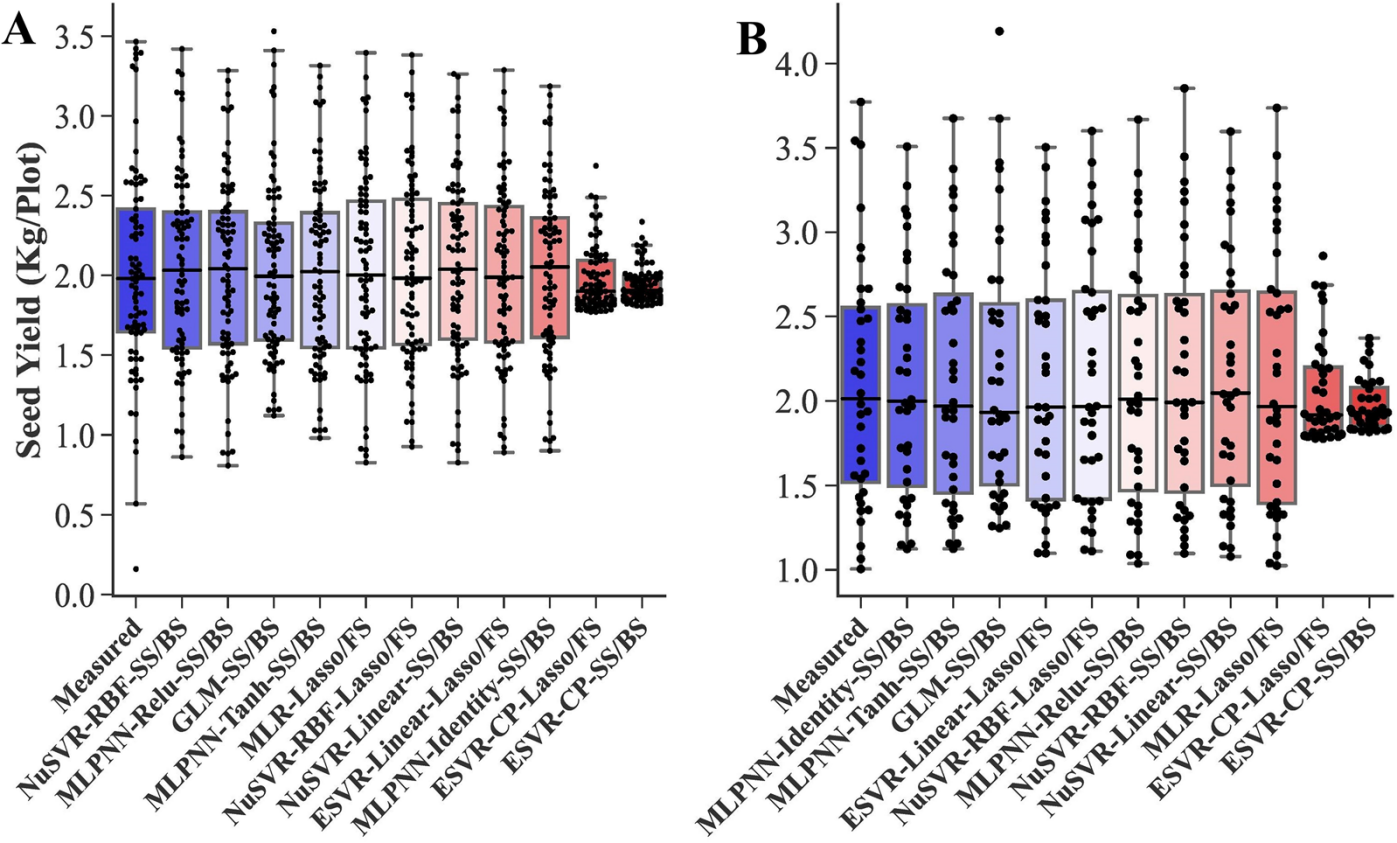
Box plots of measured and predicted SY of rapeseed using selected traits as inputs. Algorithms are sorted based on the highest to lowest R^2^ value from left to right. **A**. Training stage. **B**. Testing stage. *MLR* multiple linear regression, *GLM* generalized linear model, *NuSVR* nu-support vector regression, *ESVR* epsilon support vector regression, *MLPNN* multilayer perceptron neural network, *RBF* radial basis function, *CP* cubic polynomial, *FS* forward selection, *SS* stepwise selection, *BS* backward selection

The traits given by feature selection methods were applied to the algorithms developed in the ‘‘Seed yield prediction using all measured traits’’ Sect as inputs to estimate the power of feature selection methods and find the most compatible algorithms to predict the SY of rapeseed genotypes using fewer traits. Additional file 1 displays the performance of the algorithms using the traits obtained from each feature selection method and a summarized table has been presented in Table 5. The best training performance was seen in the NuSVR algorithm with RBF kernel function and SS/BS methods (NuSVR-RBF-SS/BS) (Fig. 8C). Also, using the same algorithm with lasso/FS methods (NuSVR-RBF-lasso/FS) resulted in the least amount of MAE in the testing dataset (Fig. 8D). The highest R^2^ value of the testing dataset was seen in the MLPNN algorithm with identity activation function and SS/BS methods (MLPNN-Identity-SS/BS) (Fig. 8B). Using SS/BS methods along with 3 algorithms including GLM and MLPNN with tanh and identity activation functions showed the least amount of testing RMSE simultaneously (Table 5). The ESVR algorithm with cubic polynomial kernel function and SS/BS methods (ESVR-CP-SS/BS) had the worst performance in all three statistical criteria of both training and testing datasets (Fig. 8E, F). A comparative box plot has been presented in Fig. 9 that shows the obvious difference between the performance of algorithms.

**Table 5:** The performance of machine learning algorithms using selected traits by feature selection methods as inputs

Algorithm	Feature selection method	Training			Testing		
		R^2^	RMSE	MAE	R^2^	RMSE	MAE
Multiple Linear Regression (MLR)	Lasso/FS	0.833	0.266	0.207	0.828	0.295	0.225
Ridge Regression (RR)	Lasso/FS	0.829	0.269	0.208	0.837	0.288	0.224
Generalized Linear Model (GLM)	SS/BS	0.834	0.265	0.212	0.842	0.283	0.225
Nu-Support Vector Regression (NuSVR)/Radial Basis Function (RBF)	SS/BS	0.845	0.256	0.200	0.830	0.293	0.228
	Lasso/FS	0.833	0.266	0.201	0.837	0.288	0.219
Epsilon Support Vector Regression (ESVR)/Linear	Lasso/FS	0.828	0.269	0.209	0.839	0.286	0.224
Epsilon Support Vector Regression (ESVR)/Sigmoid	SS/BS	0.504	0.459	0.347	0.541	0.483	0.376
Epsilon Support Vector Regression (ESVR)/Cubic Polynomial	SS/BS	0.245	0.566	0.430	0.311	0.592	0.488
	Lasso/FS	0.417	0.497	0.380	0.570	0.468	0.387
Multilayer Perceptron Neural Network (MLPNN)/Identity	SS/BS	0.827	0.270	0.219	0.843	0.283	0.224
	Lasso/FS	0.826	0.272	0.210	0.838	0.286	0.224
Multilayer Perceptron Neural Network (MLPNN)/Tanh	SS/BS	0.834	0.265	0.211	0.842	0.283	0.229
	Lasso/FS	0.828	0.269	0.208	0.839	0.286	0.224
Multilayer Perceptron Neural Network (MLPNN)/Relu	SS/BS	0.839	0.261	0.209	0.833	0.291	0.231

*FS* forward selection, *SS* stepwise selection, *BS* backward selection, *R*^2^ determination coefficient, *RMSE* root mean square error, *MAE* mean absolute error

**Fig. 10:**
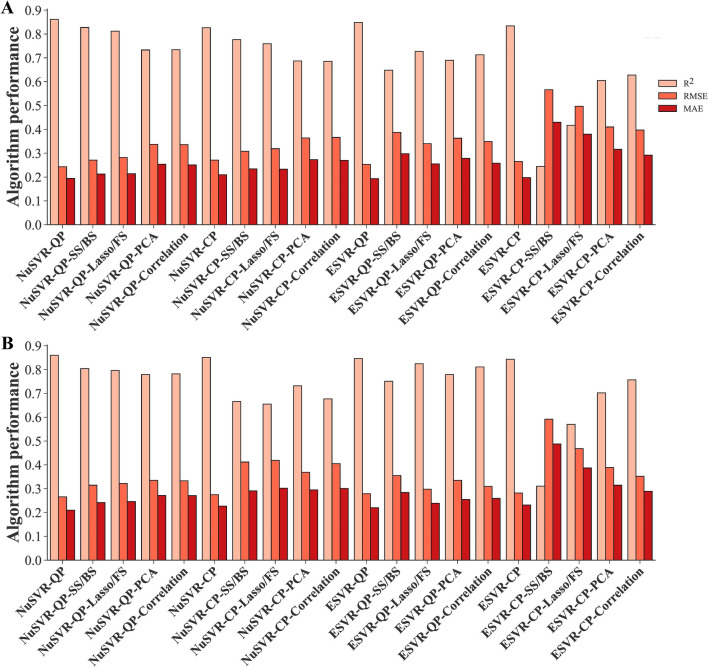
Performance comparison of NuSVR and ESVR using all measured traits and selected traits as inputs. **A**. Training stage. **B**. Testing stage. *NuSVR* nu-support vector regression, *ESVR* epsilon support vector regression, *QP* quadratic polynomial, *CP* cubic polynomial, *FS* forward selection, *SS* stepwise selection, *BS* backward selection, *PCA* principal component analysis

**Fig. 11:**
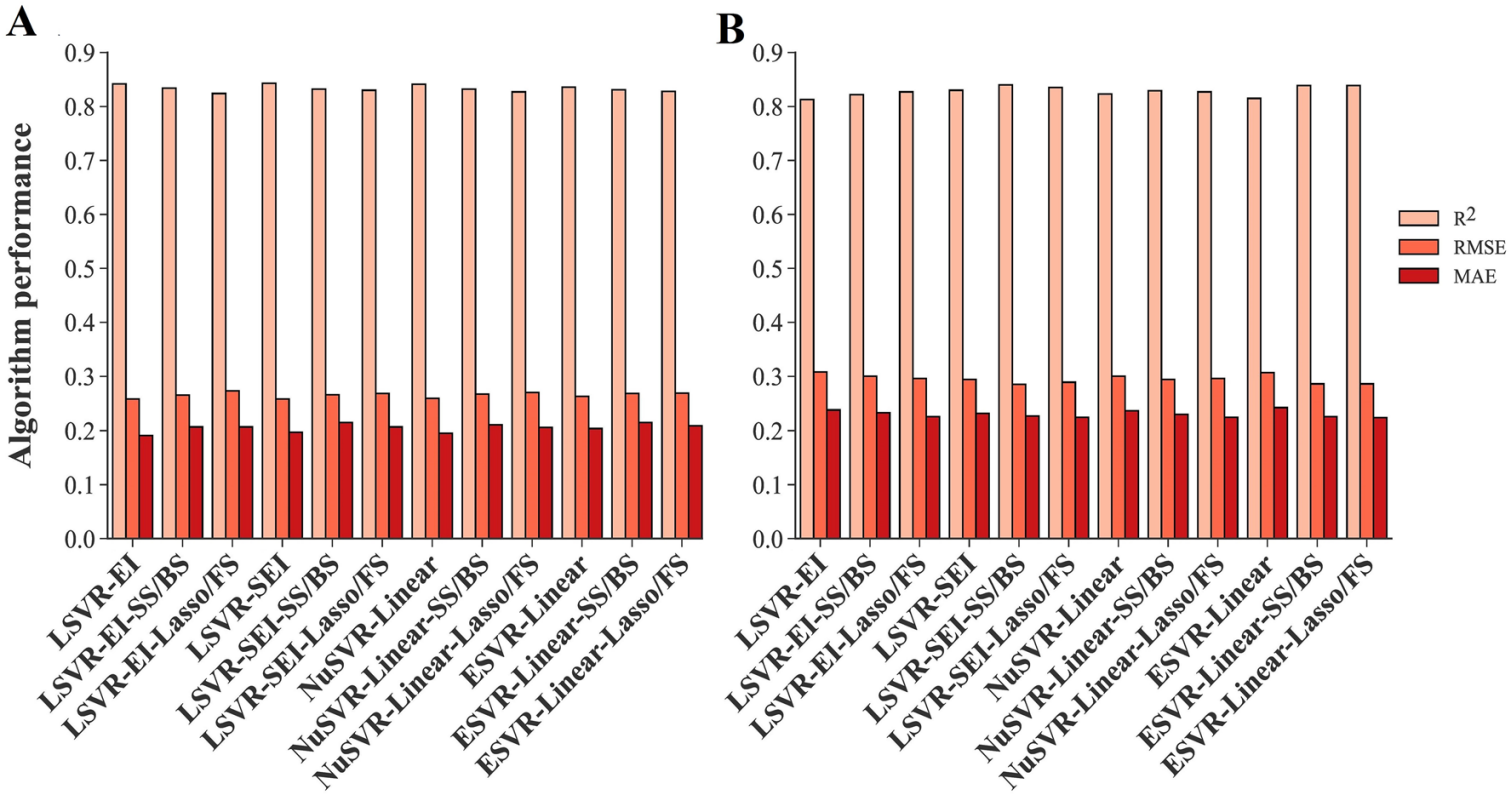
Performance comparison of SVR algorithms using all measured traits and selected traits as inputs. **A**. Training stage. **B**. Testing stage. *NuSV*R nu-support vector regression, *ESVR* epsilon support vector regression, *LSVR* linear support vector regression, *EI* epsilon insensitive, *SEI* squared epsilon insensitive, *FS* forward selection, *SS* stepwise selection, *BS* backward selection

**Fig. 12:**
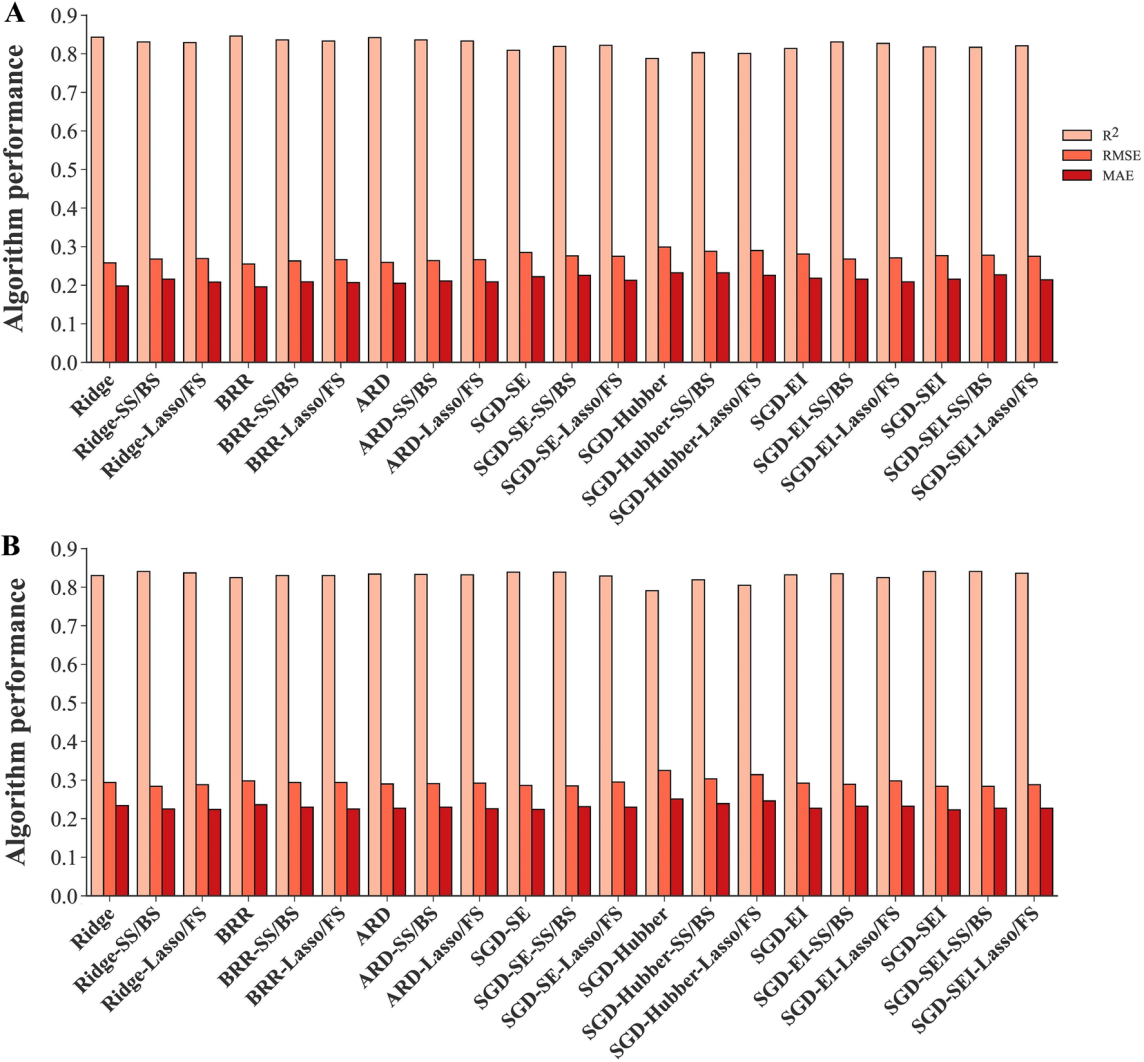
Performance comparison of regularized linear algorithms using all measured traits and selected traits as inputs. **A**. Training stage. **B**. Testing stage. *BRR* Bayesian ridge regression, *ARD* automatic relevance determination, *SGD* stochastic gradient descent, *EI* epsilon insensitive, *SEI* squared epsilon insensitive, *FS* forward selection, *SS* stepwise selection, *BS* backward selection

**Fig. 13:**
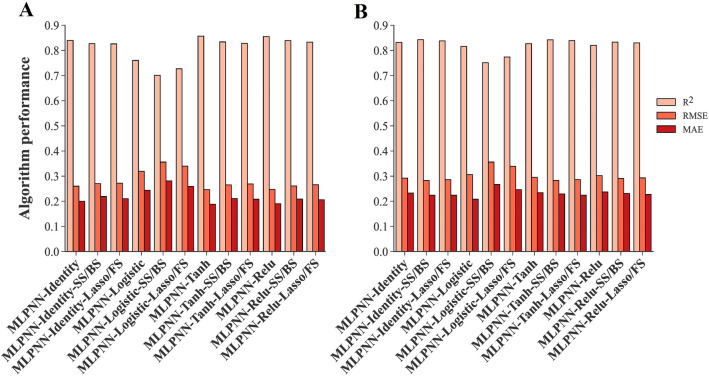
Performance comparison of MLPNN algorithms using all measured traits and selected traits as inputs. **A**. Training stage. **B**. Testing stage. *MLPNN* multilayer perceptron neural network, *FS* forward selection, *SS* stepwise selection, *BS* backward selection

Some algorithms were differentially performed using all measured traits or selected traits as inputs. For instance, NuSVR and ESVR algorithms with QP and CP kernel functions performed well when all measured traits were used as inputs; however, applying selected traits by feature selection methods led to lower performance (Fig. 10). Nevertheless, there was no noticeable difference in the performance of NuSVR and ESVR algorithms with linear kernel function, nor in LSVR algorithms when all measured traits or selected traits were applied as inputs (Fig. 11). Likewise, using all measured traits or selecting traits by feature selection methods as inputs did not significantly affect the performance of regularized linear algorithm (ridge, BRR, ADR, and SGD) (Fig. 12). Compared to using all measured traits as inputs, MLPNN algorithm with identity, tanh, and relu activation functions demonstrated better testing performance when selected traits by SS, FS, BS, and lasso methods were entered into these algorithms as inputs (Fig. 13).

In order to assess the efficiency of feature selection methods and compare them with using all measured traits as inputs to the algorithms, the mean of algorithms performance using all measured traits and selected traits by feature selection methods was calculated in both training and testing stages (Table 6). According to the results, using all measured traits as inputs to predict the SY of rapeseed genotypes resulted in highest R^2^ value and least amount of RMSE and MAE. Among the feature selection methods, the best performance in all 3 statistical criteria was achieved using the lasso and FS methods in both training and testing datasets, while PCA exhibited the worst. Moreover, based on the testing R^2^ and RMSE values, the most efficient algorithms with selected traits by correlation and PCA as inputs ranked thirty-fifth and forty-fifth among all combinations of the algorithms and feature selection methods, respectively (Additional file 1).

**Table 6:** The mean of R^2^, RMSE and MAE values of machine learning algorithms with different inputs

Inputs	Training			Testing		
	R^2^	RMSE	MAE	R^2^	RMSE	MAE
All measured traits	0.826	0.269	0.206	0.823	0.298	0.232
Selected traits by SS/BS	0.775	0.300	0.237	0.784	0.322	0.253
Selected traits by Lasso/FS	0.787	0.295	0.226	0.800	0.314	0.242
Selected traits by PCA	0.720	0.344	0.272	0.761	0.346	0.272
Selected traits by correlation	0.724	0.341	0.269	0.782	0.332	0.264

*R*^2^ determination coefficient, *RMSE* root mean square error, *MAE* mean absolute error, *FS* forward selection, *SS* stepwise selection, *BS* backward selection, *PCA* principal component analysis

### Sensitivity analysis

**Fig. 14:**
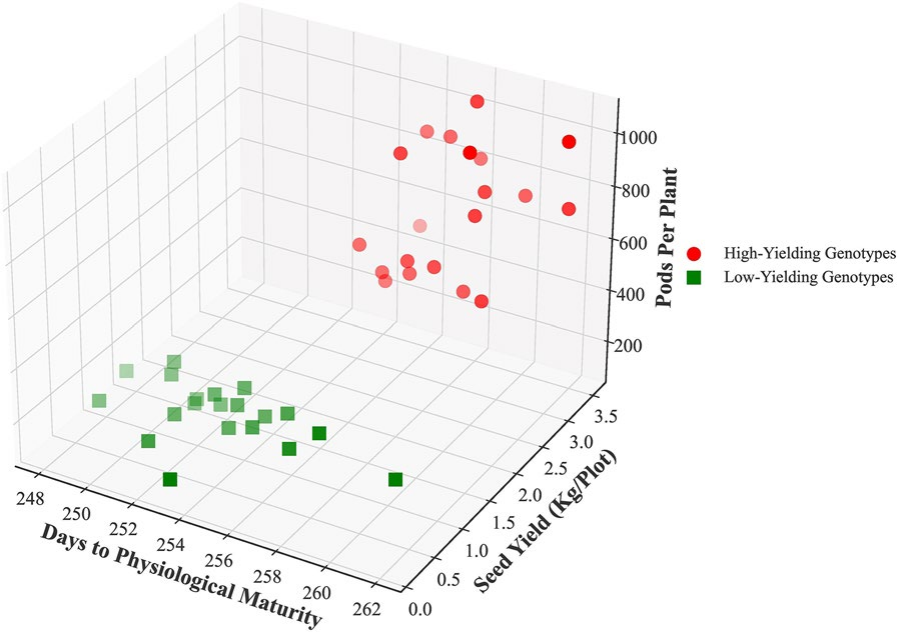
Three-dimensional figure of DPM and PP traits in high and low-yielding genotypes of rapeseed

To find the most important input traits affecting the SY of rapeseed, sensitivity analysis was conducted using the MLPNN algorithm with identity activation function, NuSVR algorithm with quadratic kernel function, and MLR algorithm. The results of sensitivity analysis showed that the highest RMSE and MAE, and the lowest R^2^ were achieved without DPM in all 3 algorithms (Table 7). The PP was also among the first 4 traits, which its elimination from the 3 algorithms caused an increase in RMSE and MAE, as well as a reduction in R^2^ value. Figure 14 shows the status of high and low-yielding genotypes from the perspective of DPM and PP traits.

**Table 7:** Sensitivity analysis of the input features on the seed yield of rapeseed

Algorithm	Eliminated trait from inputs	R^2^	RMSE	MAE
MPLNN-Identity	–	0.838	0.270	0.214
	DPM	0.804	0.297	0.231
	PMB	0.833	0.275	0.218
	PP	0.836	0.272	0.215
	PAB	0.837	0.271	0.214
	BP	0.838	0.271	0.214
NuSVR-QP	–	0.871	0.241	0.195
	DPM	0.853	0.257	0.205
	SP	0.862	0.249	0.197
	FP	0.864	0.247	0.197
	PP	0.867	0.245	0.197
	TSW	0.867	0.245	0.197
MLR	–	0.846	0.263	0.208
	DPM	0.810	0.292	0.231
	PH	0.844	0.265	0.211
	BP	0.845	0.265	0.209
	PP	0.845	0.264	0.209
	DSF	0.846	0.263	0.208

*R*^2^ determination coefficient, *RMSE* root mean square error, *MAE* mean absolute error, *MLR* multiple linear regression, *NuSVR* nu-support vector regression, *MLPNN* multilayer perceptron neural network, *QP* quadratic polynomial, *PH* plant height, *PMB* pods per main branch, *PAB* pods per axillary branches, *PP* pods per plant, *BP* branches per plant, *DSF* days to start of flowering, *DPM* days to physiological maturity, *FP* flowering period, *TSW* thousand seed weight, *SP* seeds per pod

## Discussion

Increasing SY has always been a central objective in breeding programs [12]. However, assessing SY in large populations of diverse genotypes is a laborious and time-consuming task [13, 14]. Due to the intricate interaction of genetic and environmental factors, seed yield breeding is a complex and nonlinear process [15, 16]. Consequently, breeders have adopted strategies that employ secondary traits closely associated with the primary trait to efficiently identify promising genotypes at early growth stages [17]. While conventional statistical methods have been widely used in rapeseed research to explore the relationships between SY and other traits, their assumption of linear relationships falls short in capturing the interactions and highly nonlinear associations between SY and other traits [18–22]. In contrast, the application of machine learning algorithms has proven effective in optimizing and predicting complex biological systems and, therefore, can be employed to facilitate more precise yield prediction and enhance the efficiency of breeding programs [23, 24].

### Polynomial kernels of SVR algorithms: efficient tools for SY prediction using all traits as inputs

SY is a quantitative and complex trait with a nonlinear and complicated relationship with other yield-related traits [9, 22]. Applying linear algorithms cannot fully show the relationship between SY and other traits. Using nonlinear methods such as polynomial regression can be a solution to this issue. Polynomial regression involves including polynomial terms (quadratic, cubic, etc.) in a regression equation and, as a result making new combinatorial features and allowing learning of nonlinear models [48]. However, there is a problem with polynomial regression; it is too slow and computationally intensive [35]. To address that, polynomial kernel functions in the SVR algorithms could be employed, which performs operations in the original dimension without adding any combinatorial feature and subsequently is much more computationally effective [35]. In the present study, the NuSVR and ESVR algorithms with the QP and CP kernel functions were the first four most efficient algorithms in the testing stage based on R^2^ and RMSE values (Table 1, Fig. 5B), which proved the high capability of SVR algorithms in combination with polynomial kernel functions to predict a complex trait such as SY in rapeseed.

### Hyperparameter optimization: the first approach to avoid overfitting

Overfitting is one of the major issues in the machine learning area, which occurs when an algorithm fails to generalize successfully from observed data to new data. Due to the presence of overfitting, the algorithm performs flawlessly on the training set while fitting badly on the testing set [49]. MLR and GLM algorithms with all measured traits as inputs appeared to be the most overfitted algorithm in this study (Table 1, Fig. 6A, B, E, F). Algorithm training is actually a process of hyperparameter optimization. Well-optimized parameters make a good balance between training accuracy and regularity and then inhibit the effect of overfitting. Regularization-based algorithms help us distinguish noises, meaning and meaningless features, and assign different weights to them [49–51]. In this study, MLR was the only algorithm without any hyperparameter. Hyperparameter optimization led to a better performance in the rest of the algorithms. As a result, using regularization-based algorithms with hyperparameter optimization can be a solution to overcome overfitting in rapeseed SY prediction. One of the most important advantages of these results is the reduction of required time for optimizing predictive algorithms and therefore expediting the rapeseed breeding programs.

### Feature selection

Stepwise selection is widely used to find the most important traits related to SY in plant breeding. However, discovering the best subset of the traits is an issue because all subset regression methods (SS, FS and BS) are in-sample methods for assessing and tuning models. Consequently, model selection may suffer from overfitting (fitting the noise in the data) and may not perform as well on new data [48]. To avoid this, we validated the models by using cross-validation. In accordance with the results of the SS, BS, and FS methods (Table 3), previous studies which used stepwise regression have demonstrated that pods per plant, growth duration, and pods on the main raceme [52], and pods per plant, number of branches, and duration of flowering [21] had significant effects on the SY in rapeseed genotypes. There are similarities between the result of the correlation analysis (Fig. 7) and other studies which have reported a positive and significant correlation between SY and pods per plant [18–20, 53–57], branch number [18, 55, 58, 59] and plant height [18, 54, 58] in rapeseed genotypes. Branch per plant and pods per plant were also reported as the effective traits in the first principal component associated with the yield of rapeseed accessions [19]. TSW and SP were not selected by any feature selection method and also showed a negative correlation with SY (Fig. 7). It indicates that they are not suitable indirect criteria for rapeseed SY breeding. Similar to our results, some studies reported a negative correlation between SY and TSW [15, 52, 54, 57, 59] and SP [15, 55].

**Fig. 15:**
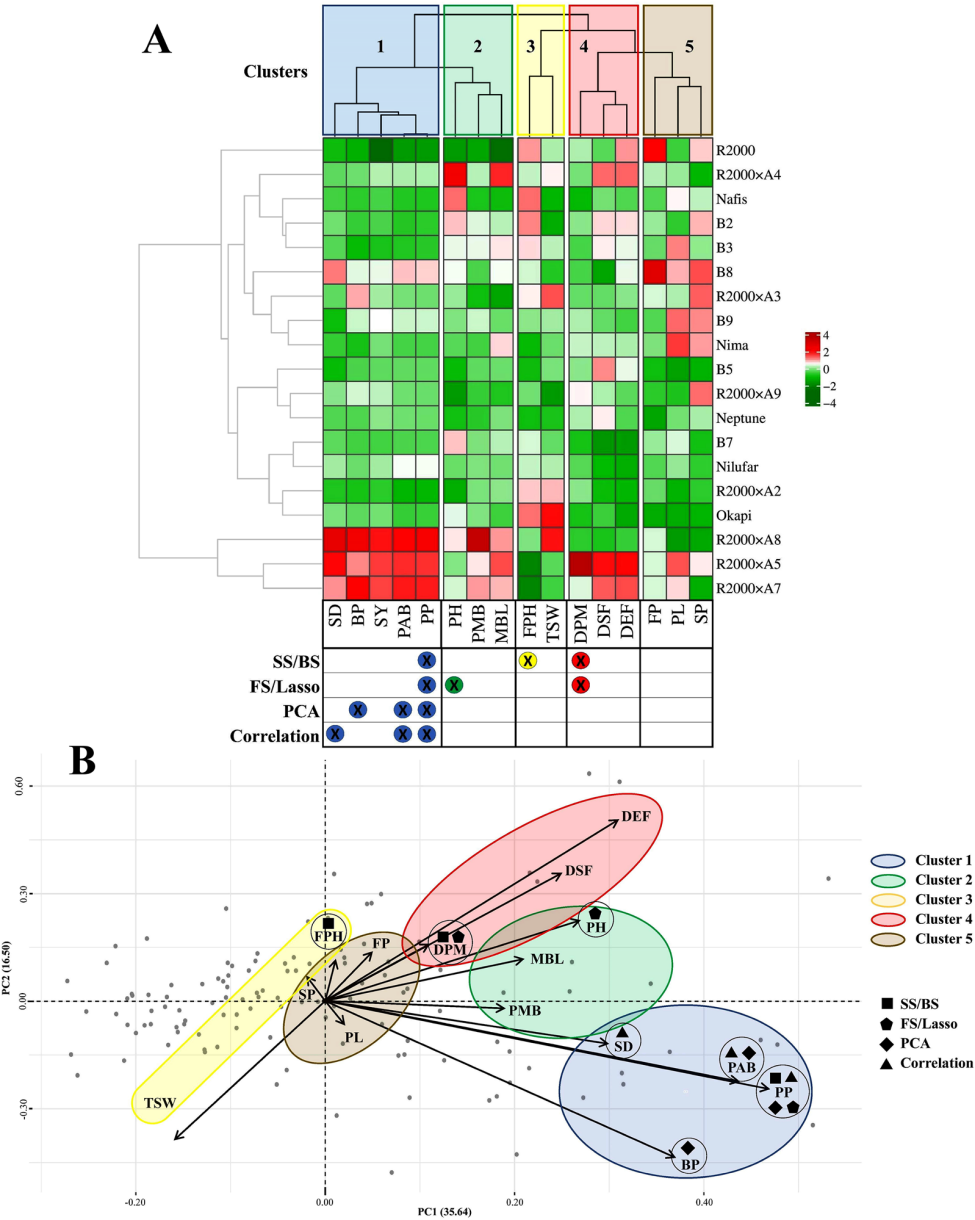
Clustering the measured traits of rapeseed genotypes using ward method. **A**. clusters demonstrated by heatmap. **B**. clusters demonstrated by PCA biplot. *PH* plant height, *PMB* pods per main branch, *PAB* pods per axillary branches, *PP* pods per plant, *BP* branches per plant, *MB*L main branch length, *FPH* first pod height from the ground, *PL* pod length, *DSF* days to start of flowering, *DEF* days to end of flowering, *DPM* days to physiological maturity, *FP* flowering period, *TSW* thousand seed weight, *SP* seeds per pod, *SD* stem diameter, *SY* seed yield, *PCA* principal component analysis, *SS* stepwise selection, *FS* forward selection, *BS* backward selection

Our findings would seem to demonstrate that correlation and PCA are not efficient methods to find proper indirect selection criteria for SY of rapeseed (Table 6). To provide a better understanding of how the traits were selected by feature selection methods, the measured traits were clustered using the Euclidean distance and ward method (Fig. 15). The results showed that all traits selected by correlation and PCA methods were in the first cluster, while SS, BS, FS, and lasso chose the traits from three different clusters, which has resulted in more efficient performance. The lack of considering the combined effects of the traits could be one of the factors that caused the inefficiency of the correlation and PCA methods. Unlike these two methods, in SS, BS, and Lasso methods, the combined effect of features is taken into account, and the combination with the best fit is chosen [35, 46].

### Feature selection: the second approach to avoid overfitting

Results from additional file 1 and Table 5 can be compared with the data in Table 1, which shows that feature selection methods could positively affect the overfitted algorithms. Compared to using all measured traits as inputs, when the traits selected by feature selection methods were applied, the amount of overfitting in the MLR algorithm was reduced, and the testing performance of the GLM algorithm dramatically improved and became among the best testing performance results which indicates an improvement in the performance of these algorithms if fewer inputs are used (Fig. 6C, D, G, H).

### Evaluating algorithms with all and selected traits: the influence of feature selection

Although using all measured traits as inputs in NuSVR and ESVR algorithms with QP and CP kernel functions led to efficient performances (Table 1, Fig. 5), applying selected traits by feature selection methods reduced their performance (Fig. 10). This revealed that the complex essence of polynomial algorithms is helpful when the data is dimensional and also nonlinear and complex relationship exists between dependent and independent variables. Nonetheless, the RBF kernel function in NuSVR and linear kernel function in ESVR showed an effective performance with selected traits by feature selection (Table 5). Therefore, one of the benefits of NuSVR and ESVR algorithms is their ability to work with different kernel functions that can provide them a flexible characteristic with different inputs. In contrast to polynomial kernel functions, no considerable difference was seen in the performance of NuSVR and ESVR algorithms with linear kernel function and also LSVR algorithms using all measured traits or selected traits as inputs (Fig. 11). Similarly, the performance of the other regularized linear algorithms (ridge, BRR, ADR and SGD) did not significantly change using all measured traits or selected traits by feature selection methods (Fig. 12). One of the major advantages of regularized linear algorithms is their ability to systematically weigh the more important features through the training process [60] and therefore, showing relatively similar performance with or without using feature selection.

The use of all measured traits as inputs to the MLPNN algorithm with identity, tanh, and relu activation functions caused overfitting of these algorithms, while the reduction of inputs by applying feature selection methods prevented overfitting or significantly reduced it (Fig. 13). Furthermore, they showed better testing performance using selected traits by SS, FS, BS, and lasso methods compared to utilizing all measured traits (Fig. 13). [61, 62] have also mentioned the crucial role of feature selection in the performance of neural networks and removing the overfitting effect. Comparing the performance of the MLPNNs with other algorithms when selected traits by feature selection methods were used, indicated that the performance of MLPNNs with fewer number of traits was more efficient than other algorithms (Table 5). Moreover, the insignificant reduction of the performance of MLPNN-Identity with traits obtained from SS and BS methods as inputs compared to the most efficient algorithm using all measured traits as inputs (NuSVR-QP) (Tables 1, 5) shows that the combination of MLPNN-Identity and SS and BS methods is an efficient approach for precise SY prediction using a much smaller number of traits (three instead of fifteen). It can greatly help breeders to effectively and simply select high-performance plants in the SY breeding programs of rapeseed since the direct selection or indirect selection via many traits for SY is practically impossible when it comes to using thousands of genotypes in a breeding program. While this paper focuses on the development of specific artificial neural networks, MLPNNs, it is important to mention that there are a diverse range of ANN algorithms beyond those presented here. Deep neural network genomic prediction (DNNGP) is a notable example, particularly in the field of plant genomic prediction, where it has been recently utilized with great success. [63].

### Indirect selection criteria

The results of sensitivity analysis (Table 7) were fully consistent with the results of feature selection since DPM and PP were the mutual traits in SS, FS, BS and lasso as the efficient feature selection methods. Rapeseed genotypes can be divided into two almost distinct groups in such a way that high-yielding genotypes has a greater number of pods per plant and longer physiological maturity time than low-yielding genotypes (Fig. 14), which is another indication that selection based on these traits can be effective in developing rapeseed varieties with higher SY performance. Comparing the results of sensitivity analysis and feature selection also indicated that DPM and PP along with PH or FPH are the most important combination traits that can greatly affect the SY of rapeseed, and as a result, can be considered as the most important indirect indicators in the breeding programs to increase rapeseed SY. Many studies have noted the direct positive effect of pods per plant on SY [19–21, 54, 59]. Increasing the number of pods per plant is the strategy that rapeseed plants employ to enhance the SY rather than improving the number or weight of seeds per pod [15]. Likewise, nitrogen availability increases the SY of rapeseed through producing more pods compared to influencing seed or pod weight [15, 64]. The direct positive effect of plant height on SY was reported by [20, 59]. This is also an indirect contribution of PP to increase the SY because a taller plant usually has more pods and thus a higher yield [18]. [65] reported that delayed maturity was a contributing factor to SY increasing, and the high potential crops for high SY had late maturity. Similarly, [18] observed a direct connection between maturity time and SY in some of their experiments.

## Conclusion

Nonlinear and complex relations between SY and yield-related traits is one of the main issues that has limited the application of conventional multivariate models to find the most effective traits for indirect selection. Regression-based machine learning algorithms along with feature selection methods, can provide a robust solution for accurate SY prediction and also introducing effective indirect selection criteria. To achieve that, different regression-based machine learning algorithms and feature selection methods were used in the present study. NuSVR and ESVR algorithms with polynomial kernel functions had the best performance when all the measured yield-related traits were used as inputs to predict the SY of rapeseed. It revealed the high potential of SVR algorithms in interpreting the nonlinear relations of dimensional data in complex biological processes. Although polynomial kernels are not proper options when fewer features are supposed to enter the SVR algorithms as inputs, RBF (with NuSVR) and linear (with ESVR) kernel functions showed effective performance with selected traits by feature selection. It showed the flexibility of NuSVR and ESVR to efficiently work with different inputs. Employing feature selection methods to find the most effective traits on the SY and using the selected features as inputs to the algorithms showed that the MLPNN algorithm with identity activation function is the most efficient and compatible algorithm with selected traits by SS and BS methods. MLPNNs are well-known and powerful algorithms, however they are sensitive to the input variables, and employing them together with proper feature selection methods would result in efficient performance. Regularized linear algorithms are effective to overcome overfitting as one of the main issues in regression and also are capable of maintaining a stable performance using numerous or selected features as inputs. According to the results of feature selection methods and sensitivity analysis, DPM, PP, and PH or FPH were the most important traits that greatly affected the SY of rapeseed. As optimizing and finding the most efficient algorithms for predicting complex biological processes is a time-consuming and challenging procedure, the optimized algorithms of this study can be used to have quicker and more efficient SY breeding programs of rapeseed, one of the most important oil crops.

## Supplementary Information

**Additional file 1: ** The performance of regression-based machine learning algorithms using selected traits by feature selection methods as inputs to predict the seed yield of rapeseed.

## Data Availability

The datasets used and/or analysed during the current study are available from the corresponding author on reasonable request.

## Abbreviations

SY: Seed yield
PH: Plant height
PMB: Pods per main branch;
PAB: Pods per axillary branches;
PP: Pods per plant
BP: Branches per plant
MBL: Main branch length
FPH: First pod height from the ground
PL: Pod length
DSF: Days to start of flowering
DEF: Days to end of flowering
DPM: Days to physiological maturity
FP: Flowering period
TSW: Thousand seed weight
SP: Seeds per pod
SD: Stem diameter
RMSE: Root mean square error
MAE: Mean absolute error
MLR: Multiple linear regression
RR: Ridge regression
BRR: Bayesian ridge regression
ARD: Automatic relevance determination
GLM: Generalized linear model
SGD: Stochastic gradient descent
NuSVR: Nu-support vector regression
ESVR: Epsilon support vector regression
LSVR: Linear support vector regression
MLPNN: Multilayer perceptron neural network
ANN: Artificial neural network
SVM: Support vector machine
SVR: Support vector regression
RBF: Radial basis function
QP: Quadratic polynomial
CP: Cubic polynomial
PCA: Principal component analysis
SS: Stepwise selection
FS: Forward selection
BS: Backward selection
